# In silico targeting of red complex bacteria virulence factors of periodontitis with β-defensin 1

**DOI:** 10.1186/s43141-022-00342-3

**Published:** 2022-04-19

**Authors:** Harini Venkata Subbiah, Polani Ramesh Babu, Usha Subbiah

**Affiliations:** 1 Human Genetics Research Centre, Sree Balaji Dental College & Hospital, Bharath Institute of Higher Education and Research, Chennai, Tamil Nadu India; 2 Center for Materials Engineering and Regenerative Medicine, Bharath Institute of Higher Education and Research, Chennai, Tamil Nadu India

**Keywords:** Antimicrobial peptide, β-Defensin 1, Virulence, Docking

## Abstract

**Background:**

Periodontitis is a multi-factorial infection with red complex bacteria playing a crucial role in the pathogenesis. As bacteria are tending to develop resistance against conventional antibiotics, new treatment modalities need to be developed. Antimicrobial peptides (AMPs) are potential tools for drug development and are gaining widespread interest. β-defensin 1 is an important AMP and forms the first-line host defense mechanism. The present study analyzed the structure and molecular docking of β-defensin 1 with the virulence factors of red complex bacteria of periodontitis. The physico-chemical properties of β-defensin 1 were determined by various online tools such as ProtParam, ProteinPredict, ToxinPred, and BioPep web servers. The structure of β-defensin 1was predicted by the SWISS-MODEL web server and the structure was evaluated by different web tools. The structure of lipopolysaccharide of *Porphyromonas gingivalis* was drawn using Chem3D ultra 11.0 software. The structure of important protein virulence factors of red complex bacteria of periodontitis was determined by the SWISS-MODEL web server. The interaction study between β-defensin 1 and virulence factors was carried out by molecular docking using Auto dock version 4.0 software and pyDock WEB server.

**Results:**

Using online tools, β-defensin 1 was predicted to be stable and non-toxic. SWISS-MODEL web server predicted Ramachandran score as 94.12% and clash score 0.0 for β-defensin 1. Auto dock version 4.0 software and pyDock WEB server analyzed the interaction to have low binding energies and hydrogen bonds were formed between the peptide and virulence factors.

**Conclusion:**

β-defensin 1 was found to have good binding interaction with the disease-causing factors of red complex bacteria of periodontitis and in turn could play a role in reducing the severity of infection. β-defensin 1 could be a potential candidate for drug development for periodontitis.

## Background

Periodontitis is a polymicrobial chronic inflammatory infection with multi-factorial causation characterized by destruction of tissues supporting the teeth (gingiva, cementum, periodontal ligament, and alveolar bone) and eventually leading to the loosening of teeth. In 1998, Dr. Sigmund Socransky classified periodontal pathogens into different clusters as the red complex, orange complex, green complex, orange-associated complex, and an Aa complex. Bacteria in the green and orange-associated clusters are early colonizers and the more pathogenic red complex bacteria are the final bacteria that colonize and lead to the destruction of the periodontium [1]. *Porphyromonas gingivalis*, *Tannerella forsythia*, and *Treponema denticola* belong to the red cluster bacteria and play a critical role in the pathogenesis of periodontitis [2]. These bacteria generally are not found alone, but together in the periodontal pockets, implicating that these bacteria work in a cooperative manner leading to the destruction of periodontal tissues. Red complex bacteria produce various virulence factors that cause the destruction of periodontal tissues and also disseminate into the bloodstream modulating host immune responses which help bacteria evade host immune responses [3]. Important virulence factors from each bacterium have been chosen for the study and explained below. *P. gingivalis* produces disease-causing factors, such as lipopolysaccharide (LPS), gingipains, fimbriae/pili, collagenase, lectins, capsule, protease, and superoxide dismutase [4]. LPS lipid A endotoxin structure produced by *P. gingivalis* can undergo acylation by two different modes- tetra-acylated lipid A designated LPS_1435/1449_ based on its molecular weights of 1435 and 1449 Da and the penta-acylated LPS designated LPS_1690_ with the molecular weight of 1690 Da [5]. Gingipains are arginine- and lysine-specific cysteine proteinases that cleave peptide and protein substrates after arginine (gingipain R) and lysine residues (gingipain K). There are two types of gingipain R, namely RgpA and RgpB and one type of gingipain K, Kgp. Gingipains degrade extracellular matrix components, including fibronectin and collagen, deregulate inflammatory responses by cleaving cytokine, immunoglobulin, and complement factors [6]. Some of the virulence factors identified in *T. forsythia* include trypsin-like proteases, sialidases SiaH, leucine-rich repeat cell-surface-associated and secreted protein bacteroides surface protein A (BspA), hemagglutinin, karilysin, and mirolysin. BspA by activating the Toll-like receptor-2 (TLR-2)-dependent pathway triggers the release of bone-resorbing proinflammatory cytokines and chemokines from monocytes and gingival epithelial cells [7]. Mirolysin and karilysin show synergistic inhibition on the complement pathway of the host system [8]. Dentilisin is one of the major virulence factors of *T. denticola* and plays a role in disease progression by degrading host cell-matrix proteins and modulate immune responses by degrading cytokines such as interleukin-1β (IL-1β) and IL-6 [9]. Cystalysin from *T.denticola* is an enzyme that is responsible for hemolytic activities and damage to the gingival and periodontal tissues [10]. Studying the mechanism of action of virulence factors of red complex bacteria and their inhibition is an important research area and would open new avenues for the treatment of periodontitis.

Antimicrobial peptides (AMPs) are important constituents of the innate immune system and have inhibitory effects against different microbes [11]. Due to the rise in resistance against conventional antibiotics, efforts are being made to bring AMPs to clinical use [12]. Defensins are small cationic AMPs that exhibit their antimicrobial activity mainly by disrupting membranes of pathogens via forming pores leading to osmotic imbalance [13]. Human β-defensin 1 is constitutively expressed in many epithelial tissues including kidney, gut, respiratory epithelium, and in the stratified epithelium of the oral cavity [14], and the present study analyzed the interaction between β-defensin 1 and virulence factors of red complex bacteria of periodontitis.

## Methods

### Computing physico-chemical properties of β-defensin 1

The FASTA sequence of β-defensin 1 was retrieved from the UniProt server (https://www.uniprot.org/uniprot/P60022) and the accession number is P60022. The amino acid sequence of β-defensin 1 was submitted to the ProtParam parameter analysis tool (https://web.expasy.org/protparam/) for the computation of various physical and chemical properties such as molecular weight, estimated half-life, instability index, and grand average of hydropathicity (GRAVY).

Other important properties of β-defensin 1 such as solvent accessibility, cell penetration, bioactivity, and toxicity were calculated using different web tools as given below. Solvent accessibility of the amino acid residues in terms of three-dimensional structure and sub- cellular localization were calculated using the ProteinPredict web server (https://predictprotein.org/). Penetration through the blood-brain barrier was also predicted using the BBPpred online server (http://bbppred.xialab.info/). The toxicity of β-defensin 1 was predicted using the ToxinPred web server (https://webs.iiitd.edu.in/raghava/toxinpred/protein.php). The antimicrobial activity was predicted by comparing the sequence of interest with the pre-existing antimicrobial peptide database AMPA (http://tcoffee.crg.cat/apps/ampa/do:ampa). The bioactivity of the peptide was predicted using the BioPep web server (http://www.uwm.edu.pl/biochemia/index.php/en/biopep).

### Homology modeling of β-defensin 1

Homology modeling of β-defensin 1 was carried using the SWISS-MODEL web server (https://swissmodel.expasy.org/interactive). Sequence ID P60022 retrieved from the UniProt server was utilized for modeling. The quality of the model was assessed and evaluated using online servers such as ERRAT, Procheck, Verify3D, and PROVE (https://saves.mbi.ucla.edu/).

### Three-dimensional structure prediction of virulence factors of red complex bacteria

Virulence factors LPS (LPS_1435/1449_ and LPS_1690_), gingipain K (Kgp), gingipain R (RgpB) of *P. gingivalis*; BspA, karilysin, mirolysin of *T. forsythia*, and cystalysin, dentilisin of *T. denticola* were selected for the study. Since the LPS structure of *P.gingivalis* was not available in PDB, tetra (LPS _1435/1449_), and penta-acylated (LPS _1690_) LPS structures of *P.gingivalis* were drawn using Chem3D ultra 11.0 software and energy minimized and cleansed using AVOGADRO software.

Protein sequences of other virulence factors of red complex bacteria were downloaded from the UNIPROT webserver and subjected to protein blast. The UNIPROT accession numbers are Gingipain K (Kgp)-P72194, Gingipain R (RgpB)-P95493, BspA-O68831, Karilysin-A0A1D3UH21, Mirolysin-A0A0F7IPS1, Cystalysin-Q56257, Dentilisin-P96091. The sequences with high similarity index were then taken into consideration and used for protein modeling. Protein modeling of different conformers was carried out using the SWISS-MODEL web server and the same were analyzed using structural assessment tool in the Swiss bioinformatics database. Conformers showing high Ramachandran plot value and low clash score were selected for further process. The Qmean value of the model was also estimated to analyze the integrity of the developed homology protein structures.

### β-Defensin 1 and virulence factors molecular docking study

#### β-Defensin 1 and LPS interaction

Docking between β-defensin 1 and LPS was carried out using Auto dock version 4.0 software. The auto grid, the component of the Auto dock, was used to compute the grid map. The best conformations search was done by adopting a genetic algorithm with the local search (GA-LS) method. The docking parameters were set to default values with 100 independent docking runs using the software Auto dock Tool Kit. Molecular graphics and visualization were performed with the UCSF Chimera package.

#### β-Defensin 1 and protein interaction

The docking between β-defensin 1 and protein virulence factors was carried out using the pyDockWEB server (https://life.bsc.es/pid/pydockweb) [15]**.** Initially, the protein complexes were prepared by carrying out protein homology modeling using SWISS-MODEL as mentioned above. Prepared models were evaluated using structural assessment by calculating the Qmean value. Models with better results were energy minimized and cleansed using protein preparation wizard (Schrodinger suite). Finally, both protein and peptide complexes were uploaded to the pyDockWEB server.

## Results

### Physico-chemical properties of β-defensin 1

The amino acid sequence of β-defensin 1 was subjected to the ProtParam protein parameter analysis tool and the data obtained is given in Table 1.

**Table 1:** Properties of β-defensin 1

Properties of β-defensin 1	Results
Number of amino acids	68
Molecular weight	7419.71
Theoretical pI	8.96
Formula	C323H513N89O93S9
Total number of atoms	1027
The estimated half-life is	30 h (mammalian reticulocytes, in vitro). > 20 h (yeast, in vivo). > 10 h (Escherichia coli, in vivo).
The instability index (II) is computed to be	32.91. This classifies the protein as stable
Grand average of hydropathicity (GRAVY):	0.157

**Fig. 1:**
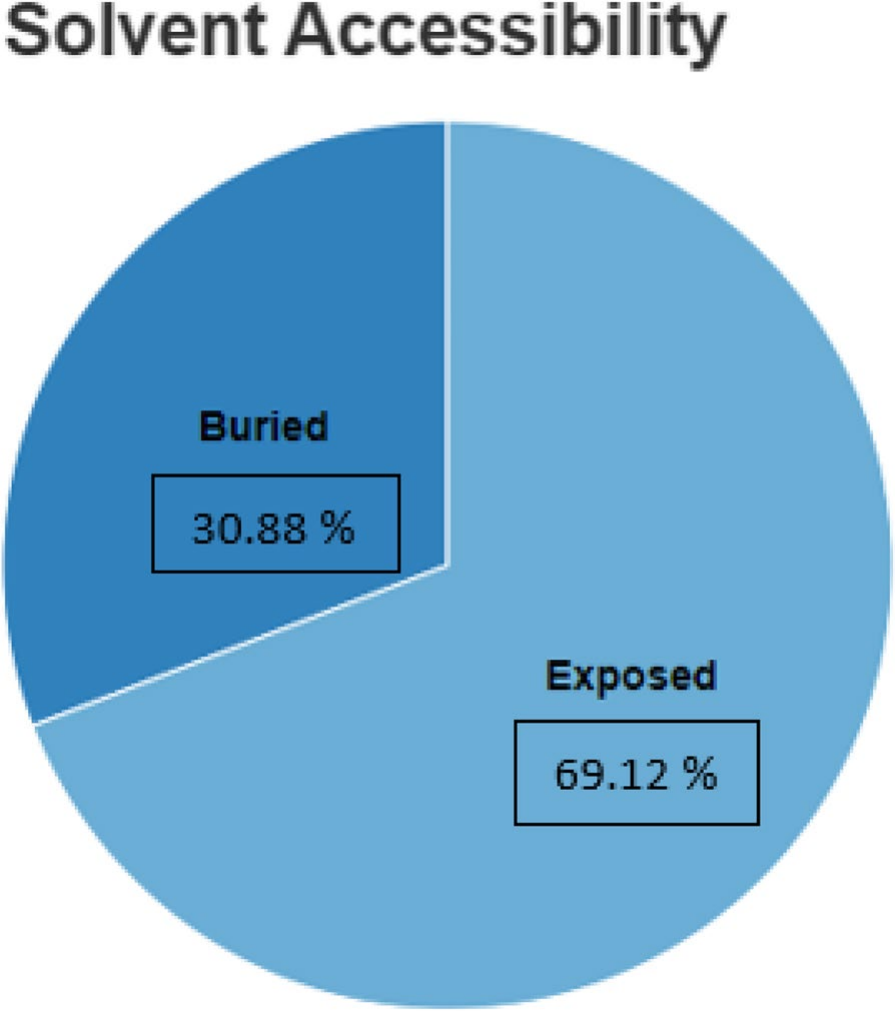
Solvent accessibility chart

Solvent accessibility of the amino acid residues was calculated using the ProteinPredict web server and the results are given below in Fig. 1. Out of 68 residues, 69.12% was found to have been exposed to solvent accessibility while 30.88% was found to be buried inside the core of the protein.

**Fig. 2:**
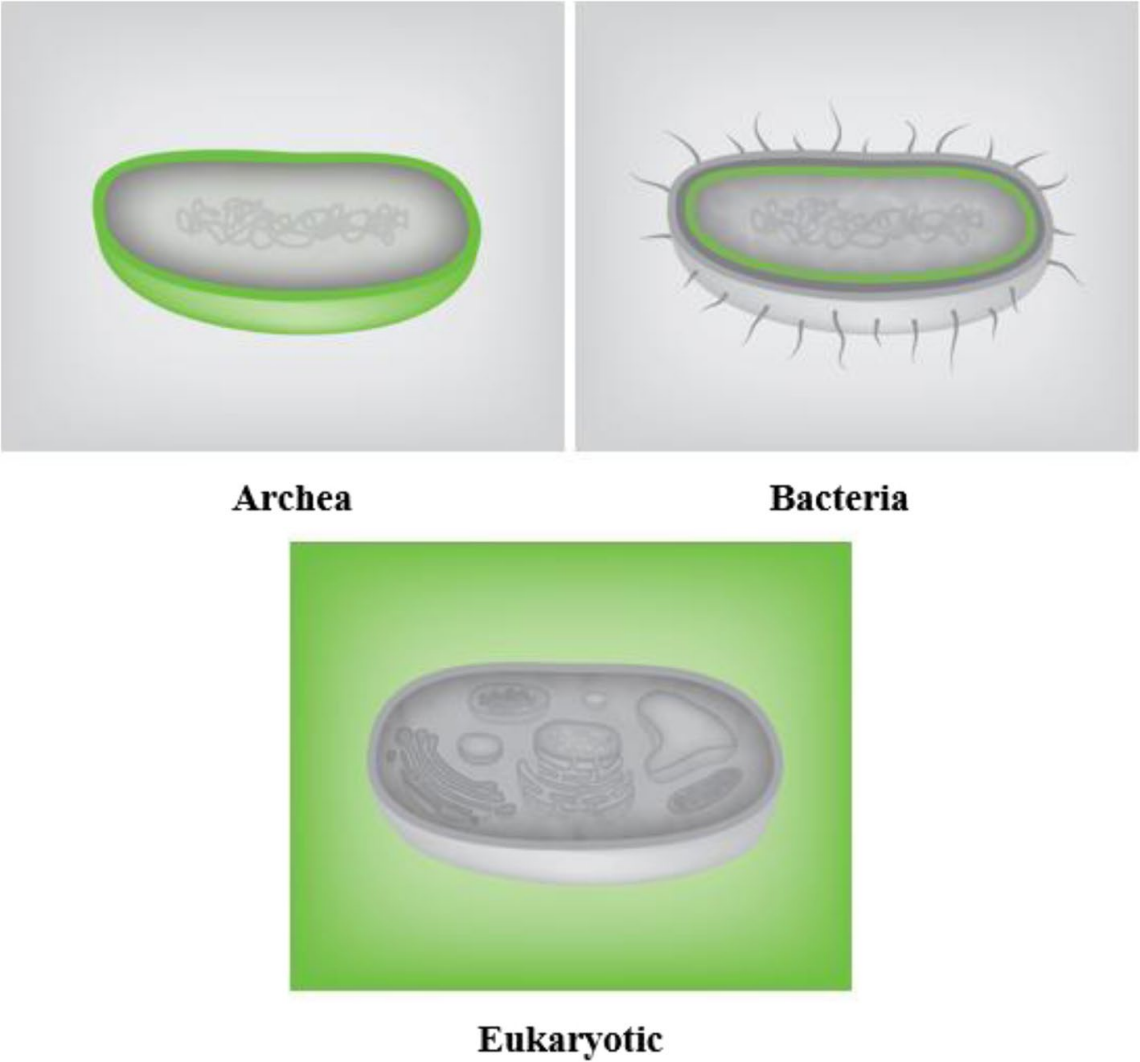
Cell penetration model of β-defensin 1

Subcellular localization of β-defensin 1 peptide, i.e., distribution and sedimentation nature of the biomolecule was studied and the results are depicted in Fig. 2. In the archaea domain, the protein was able to sediment at the cell walls of the organism whereas in the bacterial domain the protein was able to penetrate through the membrane, and finally for higher eukaryotic cells the protein was not able to penetrate even the outer cell walls. Hence, β-defensin 1 could be considered non-toxic for eukaryotic cells. Similarly, penetration through the blood-brain barrier was also predicted using the BBPpred online server and results are tabulated in Table 2.

**Table 2:** Probability of penetration of β-defensin 1 through blood-brain barrier

Uniprot Id	Probability	Class
P60022	0.376211	Non-BBP

Toxicity prediction of β-defensin 1 was carried out using the ToxinPred web server which cuts the protein into small peptides of 10 residue lengths and the prediction was carried out. The tool confirmed the non-toxic nature of the peptide.

**Fig. 3:**
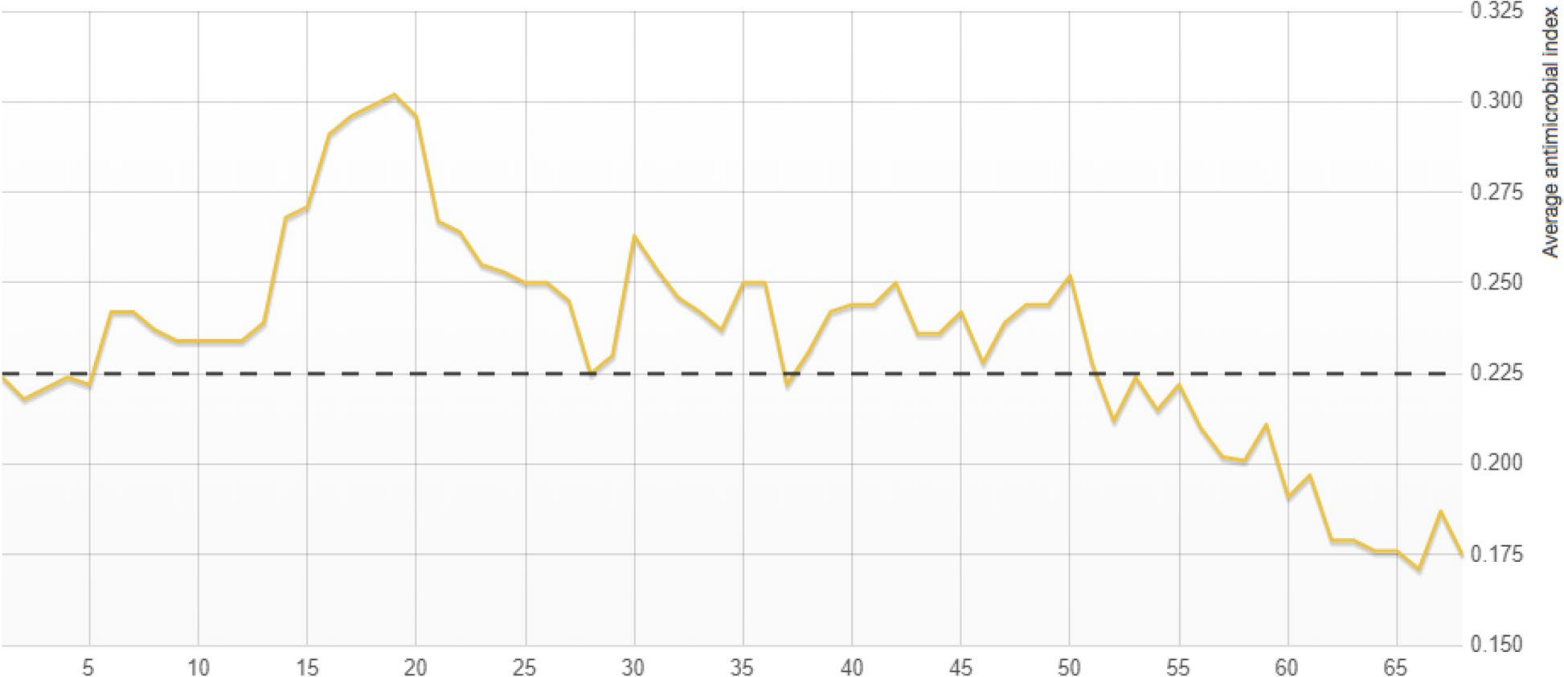
AMPA antimicrobial prediction of β-defensin 1

Antimicrobial activity was predicted by comparing the sequence of interest with the pre-existing antimicrobial peptide database and the results are shown below in Fig. 3.

Bioactivity of β-defensin 1 was predicted using the BioPep web server and predictions suggested that the peptide has excellent bioactivity toward a wide array of activity including angiotensin-converting enzyme (ACE) inhibitor, glucose uptake stimulating peptide, neuropeptide, antioxidative peptide, and anti-inflammatory peptide.

### Structure prediction of β-defensin 1by homology modeling

**Fig. 4:**
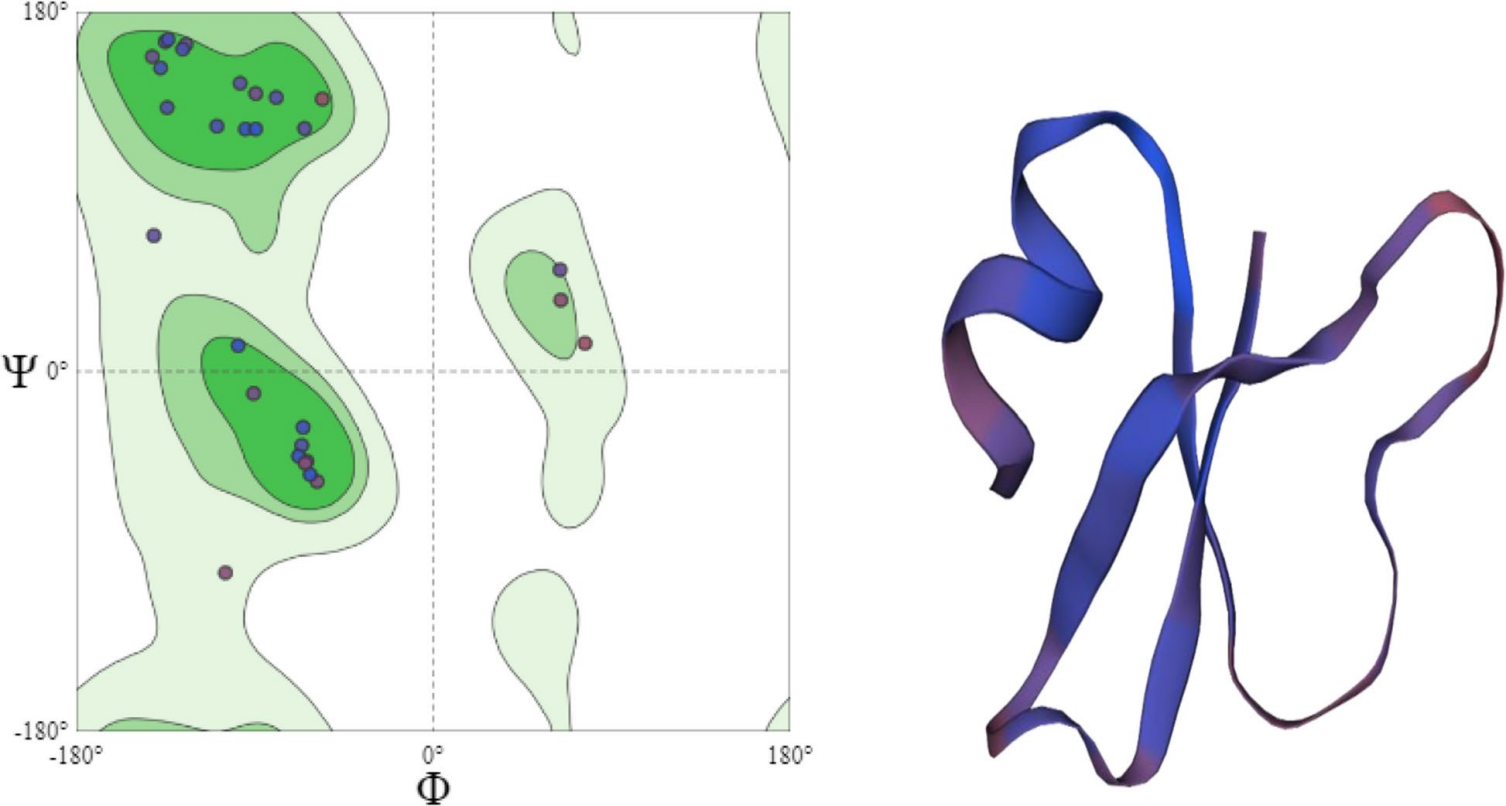
Ramachandran plot and 3D structure of β-defensin 1

The structure of β-defensin 1 was predicted by the SWISS-MODEL web server and the quality of the model was assessed and evaluated using different web tools. The results are depicted in Fig. 4 and Table 3.

**Table 3:** Homology modeling data of β-defensin 1

Isoform and model number	Ramachandran score	***Q*** mean value	Clash score	Percent identity	Mol probability score
Model_1	94.12%	0.78	0.00	97.22%	0.90

The quality of the model was assessed and evaluated using online servers such as ERRAT, Procheck, Verify3D, PROVE, and the results are tabulated in Table 4.

**Table 4:** Evaluation of model quality

ERRAT quality score	Procheck	Verify 3D	PROVE
100	4 Passes/8 Test	61.11% of the residues have averaged 3D-1D score >= 0.2	Buried outlier protein atoms total from 1 model: 0.0% Result = Pass

### Three-dimensional structures of virulence factors of red complex bacteria

**Fig. 5:**
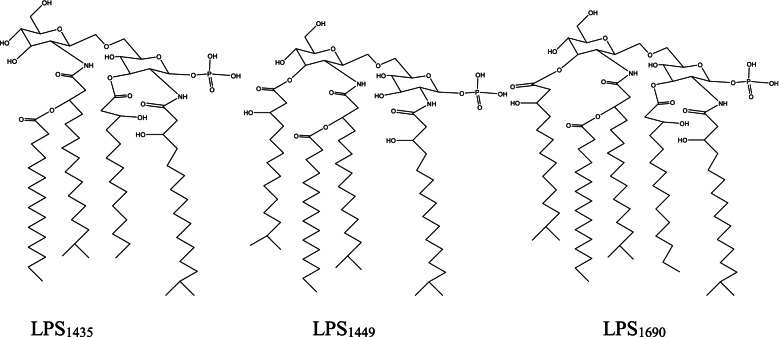
Structure of LPS of *P. gingivalis*

The structure of LPS of *P. gingivalis* is given in Fig. 5.

**Fig. 6:**
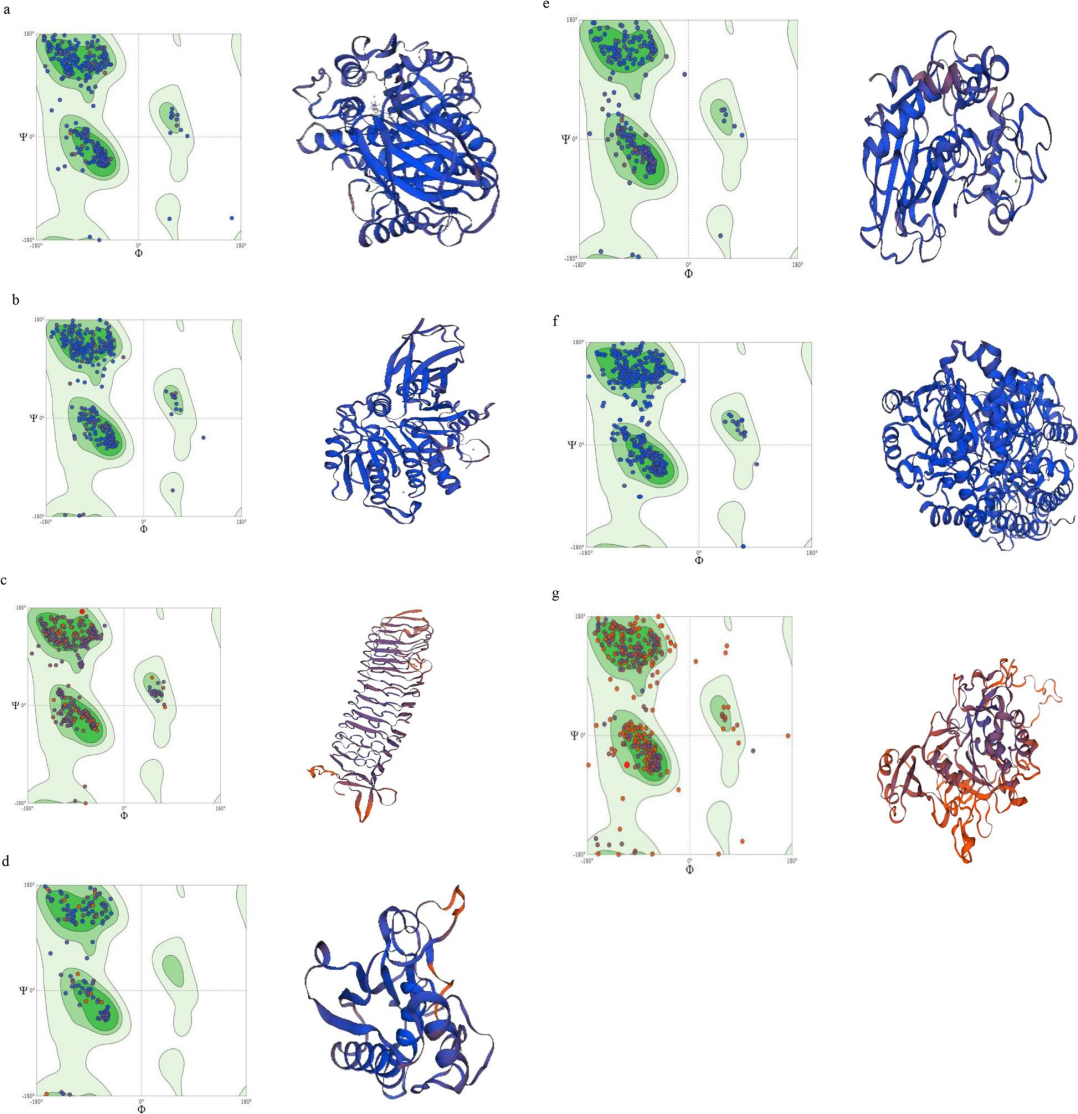
Ramachandran plots and 3D structures of virulence factors of red complex bacteria: **a** Gingipain-Kgp, **b** Gingipain-RgpB, **c** BspA, **d** Karilysin, **e** Mirolysin, **f** Cystalysin, **g** Dentilisin

The 3D structures of virulence factors are given below with their respective homology modeling scores in Fig. 6 and Table 5. Q Mean score represents quality of the protein structure predicted by SWISS-MODEL web server with a score of one being good.

**Table 5:** Model evaluation and scoring

Protein	Isoforms	Ramachandranfavored (%)	***Q*** mean (± 0.05)	Cβ	All atom	Solvation	Torsion
Gingipain Kgp	1	95.83%	0.90	− 0.97	− 0.77	− 0.62	− 0.81
Gingipain RgpB	1	97.44%	0.90	1.26	− 0.55	0.29	0.05
BspA	1	96.37%	0.68	− 0.33	− 2.70	− 2.13	− 0.69
Karilysin	1	91.72%	0.61	− 1.51	− 2.94	− 1.04	− 3.17
Mirolysin	1	95.74%	0.87	0.81	− 1.15	− 1.68	− 0.22
Cystalysin	1	96.56%	0.94	0.97	1.33	0.72	− 0.28
Dentilisin	1	85.26%	0.53	− 4.15	− 4.12	− 1.95	− 5.10

### Molecular docking of virulence factors with β-defensin 1

#### Docking of β-defensin 1 and LPS structure of P. gingivalis

**Fig. 7:**
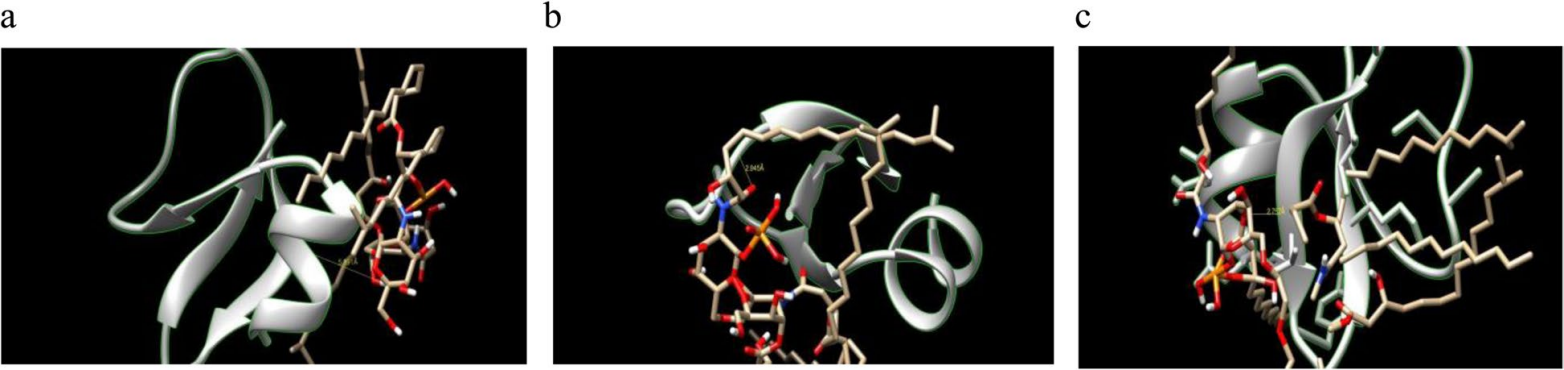
Interaction of β-defensin 1 with LPS of *P. gingivalis*: **a** With LPS_1435_, **b** LPS_1449_, and **c** LPS_1690_

The docking results of β-defensin 1 against LPS_1435/1449_ and LPS_1690_ of *P*. *gingivalis* are shown in Fig. 7 and Table 6. The binding energy (Kcal/mol), inhibition constant (Ki), and hydrogen bonds formed were used to evaluate the binding affinity of β-defensin 1 with LPS. The binding energies of LPS_1435_, LPS_1449_, and LPS_1690_ with β-defensin 1 were found to be − 7.45 Kcal/mol, − 8.31 Kcal/mol, and − 8.67 Kcal/mol respectively. LPS_1690_ had the best binding energy among the three LPS structures.

**Table 6:** Interaction results of β-defensin 1 with LPS of *P. gingivalis*

Name of biomolecule	Binding energy (Kcal/mol)	Ki	Hydrogen bond interaction		
			Hydrogen donor	Hydrogen acceptor	Bond length Å
LPS_1435_	− 7.45	452.1 μM	LPS_1435_	Gln24	5.391
LPS_1449_	− 8.31	271.6 μM	Lys22	LPS_1449_	2.945
LPS_1690_	− 8.67	321.7 μM	Thr26	LPS_1690_	2.757

#### Docking of β-defensin 1 with gingipains of P. gingivalis

**Fig. 8:**
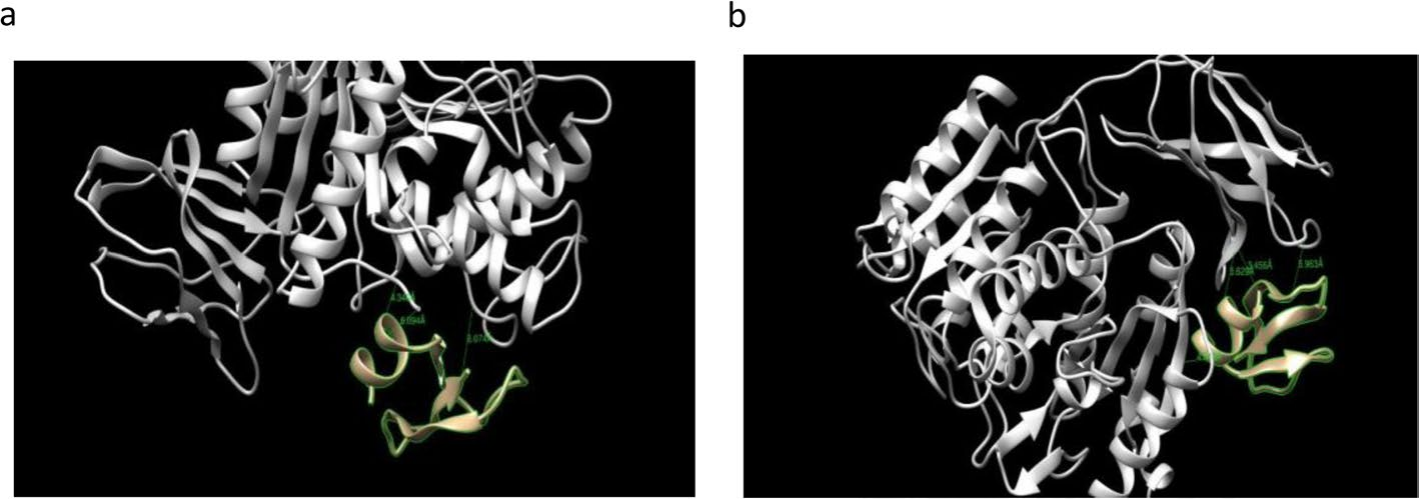
Docking of β-defensin 1 against gingipains a Kgp and b RgpB of *P. gingivalis*

The docking results of β-defensin 1 against gingipains Kgp and RgpB of *P. gingivalis* are summarized below in Fig. 8 and Table 7. β-defensin 1 formed 3 hydrogen bonds with gingipain Kgp and 4 hydrogen bonds with gingipain RgpB. The binding energy (Kcal/mol), interacting residues, and hydrogen bonds formed were used to evaluate the binding affinity of β-defensin 1 with gingipains of *P. gingivalis.* The binding energies of gingipains Kgp and RgpB with β-defensin 1 were found to be − 40.814 Kcal/mol and − 55.277 Kcal/mol. Gingipain RgpB had the best binding affinity with β-defensin 1.

**Table 7:** Interaction results of β-defensin 1 against gingipains Kgp and RgpB of *P. gingivalis*

No. of interface residues	Interface area (A^**0**^)	Binding energy (Kcal/mol)	No of hydrogen bonds	Interacting residues and bond length	No. of non- bonded contacts
17	762	− 40.814	3	Asn524 of gingipain-Kgp with Ile19 of β-defensin 1 (6.094 Å) Tyr550 of gingipain-Kgp with Val6 of β-defensin 1 (4.340 Å) Asp236 of gingipain-Kgp with Ser7 of β-defensin 1 (6.074 Å)	149
14	804	− 55.277	4	Val269 of gingipain-RgpB with Ser7 of β-defensin 1 (4.515 Å) Met617 of gingipain-RgpB with Asp1 of β-defensin 1 (3.456 Å) Ser635 of gingipain-RgpB with Cyx27 of β-defensin 1 (5.963 Å) Met617of gingipain-RgpB with Asp4 of β-defensin 1 (5.629 Å)	216

#### Docking of β-defensin 1 with virulence factors of T. forsythia

**Fig. 9:**
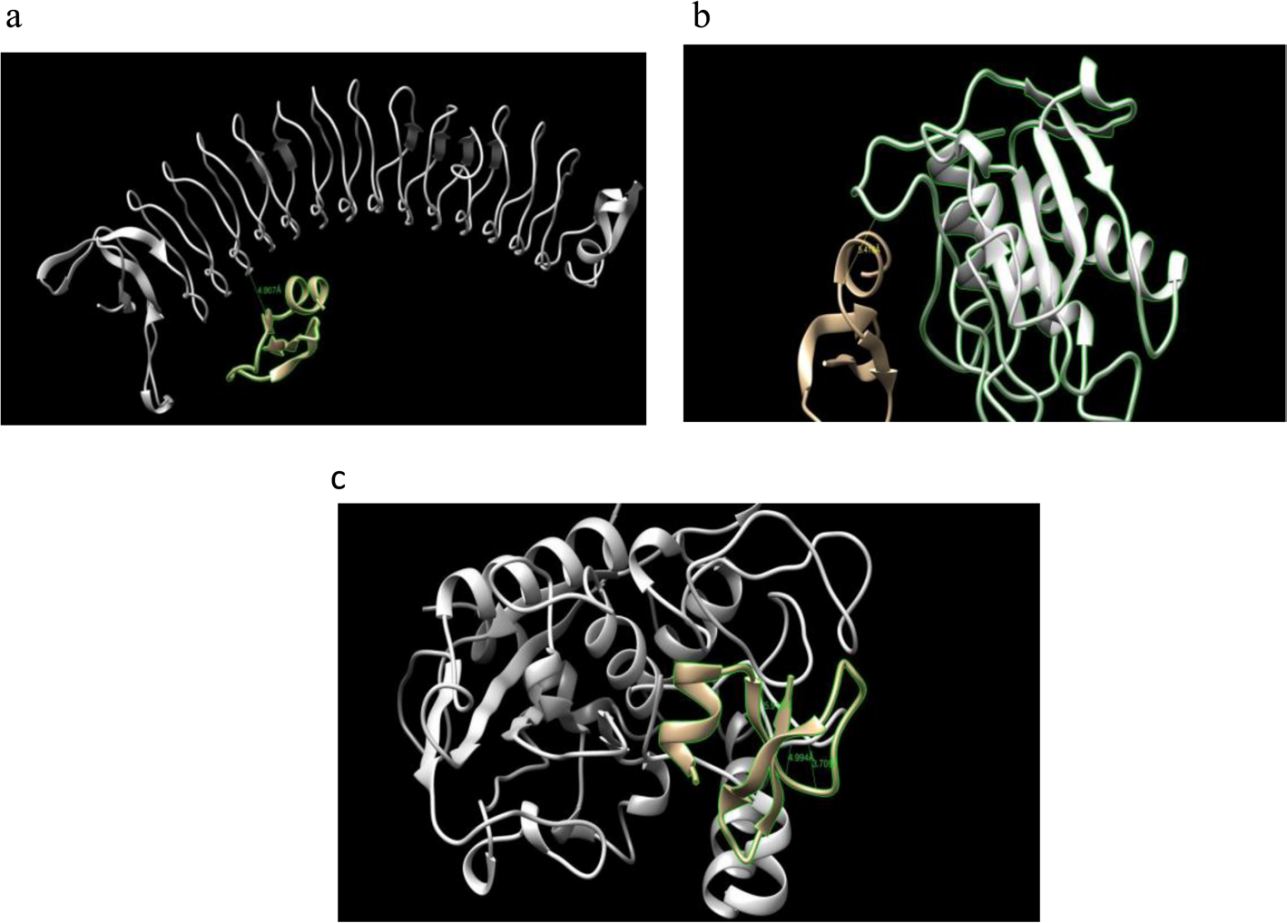
Docking results of β-defensin 1 with **a** BspA, **b** karilysin, and **c** mirolysin of *T. forsythia*

The docking results of β-defensin 1 against BspA, karilysin, and mirolysin of *T. forsythia* are summarized in Fig. 9 and Table 8. β-defensin 1interacted by forming 1 hydrogen bond with BspA, 1 hydrogen bond with karilysin, and 3 hydrogen bonds with mirolysin. The binding energies of BspA, karilysin, and mirolysin were determined to be − 40.146 Kcal/mol, − 31.705 Kcal/mol, and − 30.602 Kcal/mol. Out of the three virulence factors of *T. forsythia* studied, BspA showed the lowest binding energy and therefore had the best binding fit with β-defensin 1.

**Table 8:** Interaction results of β-defensin 1 with BspA, karilysin, and mirolysin of *T. forsythia*

No. of interface residues	Interface area (A^**0**^)	Binding energy Kcal/mol	No of hydrogen bonds	Interacting residues and bond length	No. of non-bonded contacts
9	706	− 40.146	1	Lys133 of BspA with Tyr28 of β-defensin 1 (4.907 Å)	169
9	706	− 31.705	1	Lys132 of karilysin with Tyr28 of β-defensin 1 (5.416 Å)	124
11	525	− 30.602	3	Asp239 of mirolysin with Tyr14 of β-defensin 1 (4.994 Å) Tyr258 of mirolysin with Cyx12 of β-defensin 1 (5.918 Å) Gly240 of mirolysin with Ser15 of β-defensin 1 (3.709 Å)	108

#### Docking of β-defensin 1 with virulence factors of T. denticola

**Fig. 10:**
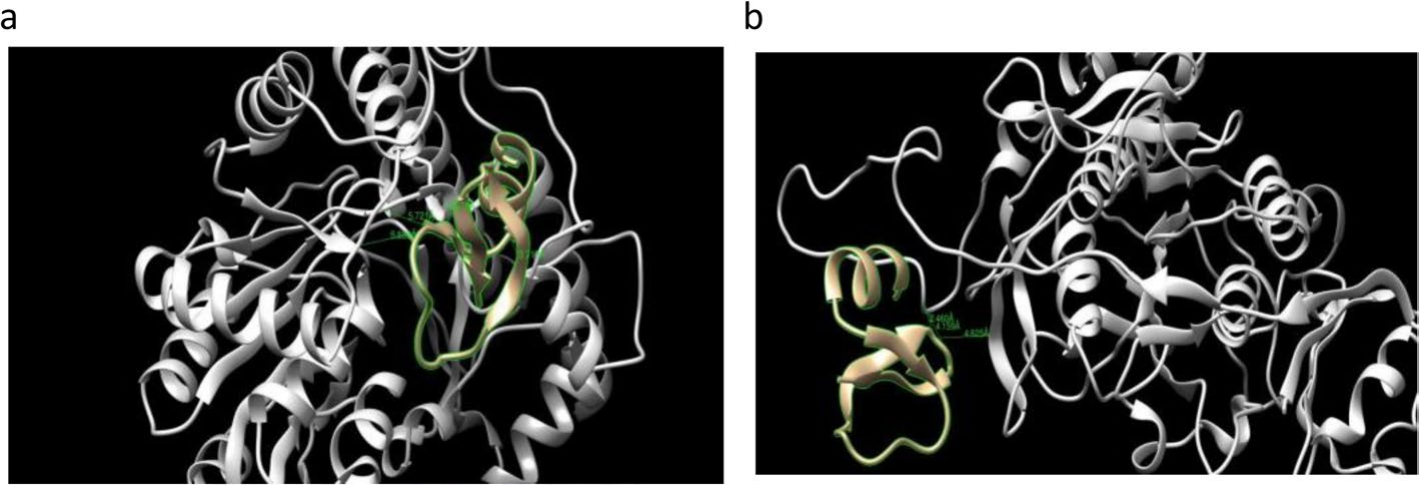
Docking results of β-defensin 1 against **a** cystalysin and **b** dentilisin of *T. denticola*

The docking results of β-defensin 1 against cystalysin and dentilisin of *T. denticola* are summarized in Fig. 10 and Table 9. β-defensin 1 interacted by forming 3 hydrogen bonds with cystalysin and dentilisin. The binding energies of cystalysin and dentilisin with β-defensin 1 were found to be − 40.846 Kcal/mol and − 41.028 Kcal/mol. Both the virulence factors of *T. denticola* showed similar binding energies.

**Table 9:** Interaction results of β-defensin 1 against cystalysin and dentilisin of *T. denticola*

No. of interface residues	Interface area (A^0^)	Binding energy Kcal/mol	No. of hydrogen bonds	Interacting residues and bond length	No. of non- bonded contacts
15	927	− 40.846	3	Thr274, Trp275 of cystalysin with Tyr28 of β-defensin 1 (5.721 Å, 5.886 Å) Leu62 of cystalysin with Tyr14 of β-defensin 1 (4.978 Å) Ser269 of cystalysin with Ser15 of β-defensin 1 (3.713 Å)	151
10	820	− 41.028	3	Asp 439 of dentilisin with Tyr14 of β-defensin 1 (4.159 Å) Tyr440 of dentilisin with Tyr14 of β-defensin 1 (2.460 Å) Pro169 of dentilisin with Arg29 of β-defensin 1 (4.625 Å)	205

The binding energies of β-defensin 1 from the docking study indicated that it had good interaction with different virulence factors of red complex bacteria of periodontitis.

## Discussion

Oral cavity provides a favorable environment for colonization by various microorganisms so oral flora requires myriad defense mechanisms in order to prevent infection. Since red complex bacteria are important periodontal pathogens, numerous research works are being carried out to study the disease pathogenesis and strategies to inhibit their virulence mechanisms. AMPs show broad-spectrum antimicrobial activity and are important constituents in the host defense against microbial challenge. They are expressed constitutively or induced in response to microorganisms. β-defensin 1 in addition to its antibacterial role performs other immune-related activities such as chemoattractants and promotes angiogenesis [16]. Studies have shown that AMPS such as lactoferrin and histatin have antibacterial efficacy against *P. gingivalis* [17, 18]. Inhibition of virulence factors could prevent or slow down the progression of periodontitis and several inhibitors from natural sources are being developed [19].

Molecular modeling plays a pivotal role in computer-aided drug design and is one of the most important virtual screening methods to study drug-receptor interaction [20]. Docking is a computational process for finding a suitable ligand that fits the protein's binding site energetically and geometrically [21]. The present study analyzed the interaction between the antimicrobial peptide β-defensin 1 and the virulence factors of red complex bacteria of periodontitis. Physico-chemical properties of β-defensin 1 were determined by the ProtParam web tool. The instability index computed by the tool predicted that the peptide was stable. The distribution nature of the peptide was studied by the ProteinPredict tool which predicted that the peptide was able to penetrate through the membrane of bacteria while it was not able to penetrate the higher eukaryotic membrane. β-defensin 1 can exert its antimicrobial effects without harming normal eukaryotic cells due to the positive charge on the peptide which can interact with negatively charged membranes of microbes, while the membrane composition of eukaryotic cells is mainly uncharged zwitterion (neutral) phospholipids, sphingomyelins, and cholesterol and hence cannot affect eukaryotic cells [22].

The structure of β-defensin 1 was predicted by the SWISS-MODEL web server which gave the Ramachandran score of 94.12% and clash score of 0.0. The 3D structures of virulence factors of red complex bacteria of periodontitis were predicted by the SWISS-MODEL server. The structure of LPS of *P.gingivalis* was drawn by Chem3D ultra 11.0 software.

Docking of β-defensin 1 with LPS of *P. gingivalis* was studied by Autodock version 4.0 software. Docking of β-defensin 1 with other virulence factors was carried out by pyDockWEB server. Docking studies with AutoDock software and pyDockWEB server revealed that β-defensin 1 was able to bind with low docking energies indicating their high affinity with the selected proteins of bacteria. β-defensin 1 showed significant interaction with the virulence factors and could play a role in the inhibition of pathogenesis of periodontitis mediated by red complex bacteria.

## Conclusion

β-defensin 1 is one of the important components of host’s natural innate immunity and in this study, through molecular docking, it has been found that β-defensin 1 interacted with various virulence factors of periodontitis which suggests that it could be used as an adjunct/alternative to antibiotics for the treatment of periodontitis. Through various online tools, β-defensin 1 was found to be non-toxic. It could be a promising candidate in the treatment of periodontitis and has a great potential application in the drug development process.

## Data Availability

All data analyzed during this study are included in this article.

## Abbreviations

ACE: Angiotensin-converting enzyme
AMP: Antimicrobial peptide
AMPA: Antimicrobial peptide database
BspA: Bacteroides surface protein A
GRAVY: Grand average of hydropathicity
IL-1β: Interleukin-1β
LPS: Lipopolysaccharide
TLR-2: Toll-like receptor-2
